# METTL3 modulates m6A modification of CDC25B and promotes head and neck squamous cell carcinoma malignant progression

**DOI:** 10.1186/s40164-022-00256-3

**Published:** 2022-03-14

**Authors:** Yu-qing Guo, Qiang Wang, Jun-guo Wang, Ya-jun Gu, Pan-pan Song, Shou-yu Wang, Xiao-yun Qian, Xia Gao

**Affiliations:** 1 Medical School of Nanjing University, Nanjing, 210093 Jiangsu China; 2 Department of Hepatobiliary Surgery, Affiliated Drum Tower Hospital, Medical School of Nanjing University, Nanjing, 210008 China; 3 Department of Otolaryngology Head and Neck Surgery, Affiliated Drum Tower Hospital, Medical School of Nanjing University, Jiangsu Provincial Key Medical Discipline (Laboratory), Nanjing, 210008 China; 4 Research Institute of Otolaryngology, Nanjing, 210008 China; 5 Jiangsu Key Laboratory of Molecular Medicine, Medical School of Nanjing University, Nanjing, 210093 China; 6 Department of Otolaryngology Head and Neck Surgery, Affiliated Drum Tower Hospital, Medical School of Nanjing University, No.321 Zhongshan Road, Nanjing, 210008 Jiangsu China

**Keywords:** m6A, METTL3, CDC25B, Cell cycle, HNSCC

## Abstract

**Background:**

N6-methyladenosine (m6A) RNA methylation and its methyltransferase METTL3 have been widely reported to be involved in different cancers by regulating RNA metabolism and function. Here, we aimed to explore the biological function and clinical significance of m6A modification and METTL3 in head and neck squamous cell carcinoma (HNSCC).

**Methods:**

The prognostic value of METTL3 expression was evaluated using tissue microarray and immunohistochemical staining analyses in a human HNSCC cohort. The biological role and mechanism of METTL3 in HNSCC tumour growth, metastasis and angiogenesis were determined in vitro and in vivo.

**Results:**

M6A levels and METTL3 expressions in HNSCC tissues were significantly increased compared with paired adjacent tissues. Meanwhile, METTL3 was an independent risk factor for the prognosis of HNSCC patients. Moreover, METTL3 overexpression promoted HNSCC cell proliferation, migration, invasion, and angiogenesis, while knockdown of METTL3 had an opposite effect in vivo and in vitro. Mechanistically, METTL3 enhanced the m6A modification of CDC25B mRNA, which maintained its stability and upregulated its expression, thereby activating G2/M phase of cell cycle and leading to HNSCC malignant progression.

**Conclusions:**

METTL3 may be a potential prognostic biomarker and therapeutic target for HNSCC.

**Supplementary Information:**

The online version contains supplementary material available at 10.1186/s40164-022-00256-3.

## Introduction

Head and neck tumor is the sixth most common cancer in the world, including the malignant tumors in the oral cavity, oropharynx, hypopharynx, and pharynx. Of all, head and neck squamous cell carcinoma (HNSCC) accounts for more than 90% of the most common histological type of head and neck tumor[1, 2]. New cases of HNSCC exceed 1,000,000 each year, with 543,000 death around the world [3]. Smoking and drinking have been proved to be the most important risk factors for HNSCC [4, 5]. Meanwhile, it also showed that human papillomavirus (HPV) is a strong independent risk factor for HNSCC [6]. Currently, surgery, radiotherapy, and/or chemotherapy are the conventional treatment for HNSCC. Although there are some improvements for HNSCC therapy, the outcome of patients with HNSCC remains poor, especially for patients with advanced HNSCC. The 5-year survival rate for HNSCC is around 50%, with little improvement in the past 20 years [7]. This may be closely related to late diagnosis, low treatment response, cancer recurrence, and high metastasis rate [8].

It has been widely reported that RNA modification and RNA-binding proteins could affect abnormal expression of oncogene and tumor suppressor via regulation of RNA metabolism and function, leading to tumor initiation and development. RNA-binding protein mainly regulates RNA post-transcriptional process, including regulating RNA splicing, polyadenylation, mRNA stability, mRNA localization, and translation [9]. N6-methyladenosine (m6A) is one of the most abundant RNA modifications in eukaryote RNA, which accounts for about 50% of total modifications [10, 11]. It has been reported that m6A modification usually enriches around stop codon and 3’-UTR region, and participates in each period of the RNA metabolism [12–14]. M6A modification is mainly controlled by methyltransferase and demethylase (also called writer and eraser, respectively); m6A methyltransferases enhances the m6A modification of RNAs and affect multiple signal pathways in cancer biology. Targeting m6A methyltransferases have shown promising clinical value for cancer therapy [15]; and m6A binding protein (also called reader) could bind to the m6A motif to regulate RNA function [14, 16], including RNA splicing, stability, transport, localization, and translation, which has been reported to play a critical role in multiple biological processed like tumorigenesis and hematopoiesis [17, 18].

METTL3 is the first identified and acts as the major catalytic component of the m6A methyltransferase complex. The biological functions of METTL3 have been widely studied to be involved in different aspects of cancer development. With the development of high-throughput sequencing and detection technology, lots of m6A modification-related RNAs have been found to be the potential cancer biomarkers and therapeutic targets [19, 20]. Lots of studies have showed that abnormal expression of METTL3 in acute myeloid leukemia (AML) [21, 22], gastric cancer [23, 24], pancreatic cancer [25], breast cancer [26], hepatocellular carcinoma [27], and non-small cell lung cancer (NSCLC) [28] and is associated with nasopharyngeal carcinoma [29], and ovarian cancer [30]. It also reported that METTL3 could increase YAP mRNA stability and translation, and promote NSCLC metastasis and therapy resistance [31]. However, the role of METTL3 in HNSCC field is unclear, which pushes us to explore its function in HNSCC development.

CDC25 is a highly conserved phosphatase with dual serine and threonine specificity, which consists of 3 subtypes, CDC25A, CDC25B, and CDC25C. The N terminal region of CDC25 has phosphorylation and ubiquitination site to regulate phosphatase activity, while the C terminal region contains the catalytic site [32]. CDC25B dephosphorylates and activates cyclin-dependent kinase/cyclin complex (CDK1/Cyclin B), which is responsible for cell cycle G2-M transition [33–36]. It has reported that CDC25B inhibitors (vitamin K analogs like adociaquinone B [37], NSC 663284 [38], and menadione [39].) could inhibit CDC25B diphosphatase activity and suppress tumor progression. However, the function of CDC25B in HNSCC remains elusive.

In this study, we revealed that METTL3 could mediate m6A modification on CDC25B mRNA and promote the malignant progression of HNSCC, proposing that METTL3 may be a potential predictive biomarker and therapeutic target for HNSCC.

## Materials and methods

### Patients and specimens

All HNSCC and paracancerous normal frozen tissues were collected from the Affiliated Drum Tower Hospital, Medical School of Nanjing University (Nanjing, Jiangsu, China), including 5 pairs of HNSCC with paracancerous normal tissues and 100 HNSCC tissues used for tissue microarray. The selection criteria were as follows: patients diagnosed with primary HNSCC who have not received any treatment other than surgical resection from 2013 to 2017. They were used for qRT-PCR, western blotting, dot blotting, and IHC assay. The Nanjing Drum Tower Hospital Ethics Committee has endorsed this study.

### Cell culture and transfection

Human HNSCC cell lines (SAS, FaDu, Hep2, Tu212, and Tu686) were purchased from Yaji Biotechnology (Shanghai, China). Human HUVEC cell was obtained from the Type Culture Collection of the Chinese Academy of Sciences (Shanghai, China). HUVEC, SAS, FaDu, Tu212, and Tu686 cell lines were cultured in RPMI-1640 medium. Hep2 cell was cultured in DMEM medium. siRNA, plasmid transfection, and viral transduction were performed as previously reported[23].

### shRNA, plasmid, and siRNA construct

All shRNA and plasmid were constructed as our previous study[23]. METTL3 shRNA, METTL3 cDNA were cloned into lentiviral vectors. CDC25B siRNAs were obtained from RiboBio (Guangzhou, China). All sequences can be seen in the Additional file 1: Table S2.

### Tissue microarray (TMA) and Immunochemistry staining

The HNSCC TMA were made in the Department of Pathology in Nanjing Drum Tower Hospital. IHC on tissue sections was carried out as previously described [23]. Scoring was conducted independently by two pathologists with the semi-quantitative immunoreactivity score (IRS) standard as previously reported [23]. The final score was designated as low or high expression group using IRS: low expression was defined as an IRS of 0–6, and high expression as an IRS of 8–12.

### Human m6A-mRNA epitranscriptomic microarray

Total RNA was extracted from METTL3 knockdown SAS cells and the corresponding control cells, and was incubated with an anti-m6A antibody and magnetic beads for immunoprecipitation. After eluting, we tagged m6A modified RNA as “IP” and labeled with Cy5, and unmodified RNA in the supernatant as “Sup” and labeled as Cy3, they were then used as cRNAs in analysis using Arraystar Super RNA Labeling Kit. After hybridizing these cRNAs onto a Arraystar Epitranscriptomic Microarray slide (8 × 60 K, Arraystar) and washing it, an Agilent Scanner G2505C was used to scan the array. A normalization was done on the raw intensities of Cy5-labeled “IP” and Cy3-labeled “Sup” using an average of log2-scaled Spike-in RNA intensities. These intensities gave the basis to calculate m6A methylation level and quantity. Differentially m6A-methylated RNAs were filtered by fold change > 1.2 and p-value < 0.05, and then be used for hierarchical clustering, GO analysis and pathway analysis.

### Dot blot assay

The dot blot assay was conducted as previously described [23]. mRNA was extracted and denatured. After adding mRNA to a Hybond-N + membrane, the membrane was crosslinked, washed, blocked in blocking buffer, and incubated with an anti-m6A antibody. The membrane was then washed and incubated with an anti-mouse antibody. After washing, Hyperfilm ECL was added onto the membrane and then images were acquired. The loading control was constructed with methylene blue interacting with mRNA.

### The mRNA stability assay

The actinomycin D (MCE, San Dimas, CA, USA) was used to inhibit RNA synthesis. The concentration used was 5 µg/mL. Cells were collected at different time points (0, 4 or 8 h) after actinomycin D treatment. Total RNA was then extracted and analyzed by qRT-PCR. The remaining RNA levels at each time point were normalized to the level at the beginning (0 h).

### Western blot assay and Quantitative real-time RT-PCR (qRT-PCR)

Western blot assay and qRT-PCR were performed as previously described[23]. The primers and antibodies were listed in Additional file 1: Tables S2 and S3.

### Proliferation and cell cycle assay

For the colony formation assay, HNSCC cells were seeded in 12-well plates (500 cells per well) and incubated in a carbon dioxide cell incubator for 8–12 days. Cells were then fixed with methanol and stained with crystal violet (Beyotime) for 30 min each.

For the CCK8 assay, 1000 cells were plated in 96-well plates for 24, 48, 72, 96, and 120 h before determining cell viability using the manufacturer's protocol (Dojindo, Kumamoto, Japan). When adding menadione, different concentrations were added in wells after cell adhesion (about 6 h).

The cell cycle assay was performed according to the Cell Cycle Staining Kit protocol (MultiScience, Hangzhou, China). After collecting 2 × 10^5^–1 × 10^6^ cells, 1 ml DNA staining solution and 10 μl permeabilization solution were added and then vortexed for 5-10 s. Cell cycle phases were detected on a flow cytometer after 30 min incubation in dark at room temperature.

### Transwell assay

Transwell assay was performed as previously described [23]. Briefly, after coating the upper transwell chambers with or without Matrigel (BD, Bioscience), HNSCC cells were seeded with serum-free medium, and medium containing 10%FBS was added in the lower chambers. Chambers were incubated, fixed with paraformaldehyde, stained crystal violet, and then imaged.

### Animal study

BALB/c male nude mice (5–6 weeks old) were purchased from Nanjing Biomedical Research Institute of Nanjing University (Nanjing, Jiangsu, China) and maintained in SPF facilities. Tumour xenograft models were established in nude mice bearing: SAS cells cells stably transfected with METTL3-shRNA and the corresponding control vector. The different HNSCC cells (5 × 10^6^) were subcutaneously injected into the right axilla of nude mice (n = 6 per group). Tumour volume was monitored every other day (volume = length × width^2^ × 1/2). At the end of the experiment, the mice were sacrificed, and the tumors were weighed and imaged and were then fixed in 4% paraformaldehyde or frozen for further analyses.

### HUVEC tube formation assay

HUVEC tube formation assay was conducted as previously reported [23]. HNSCC cells were cultured and the conditioned medium was mixed with HUVEC cells in 96-well plates coated with Matrigel™(BD Biosciences). After incubating for 24 h, images were taken and 3 random fields were used to count tubes.

### Statistical analysis

Pearson Chi-Square test was used to evaluate IHC scores of HNSCC and paracancerous normal tissues, as well as METTL3 expression with clinicopathological features. Wilcoxon test (grouped) was used to assess IRS differences for the METTL3 high and low expression group. Kaplan–Meier curve with a low-rank test was used to analyze survival differences. The two-tailed Student’s t-test and one-way ANOVA test were used to compare difference significance between 2 and 3 groups. P-value < 0.05 was considered statistically significant. All statistical analyses were performed using Prism 6 (GraphPad Software Inc., La Jolla, CA) and R software (version 2.10.1).

## Results

### High expression of METTL3 in HNSCC correlates with poor prognosis

**Fig. 1:**
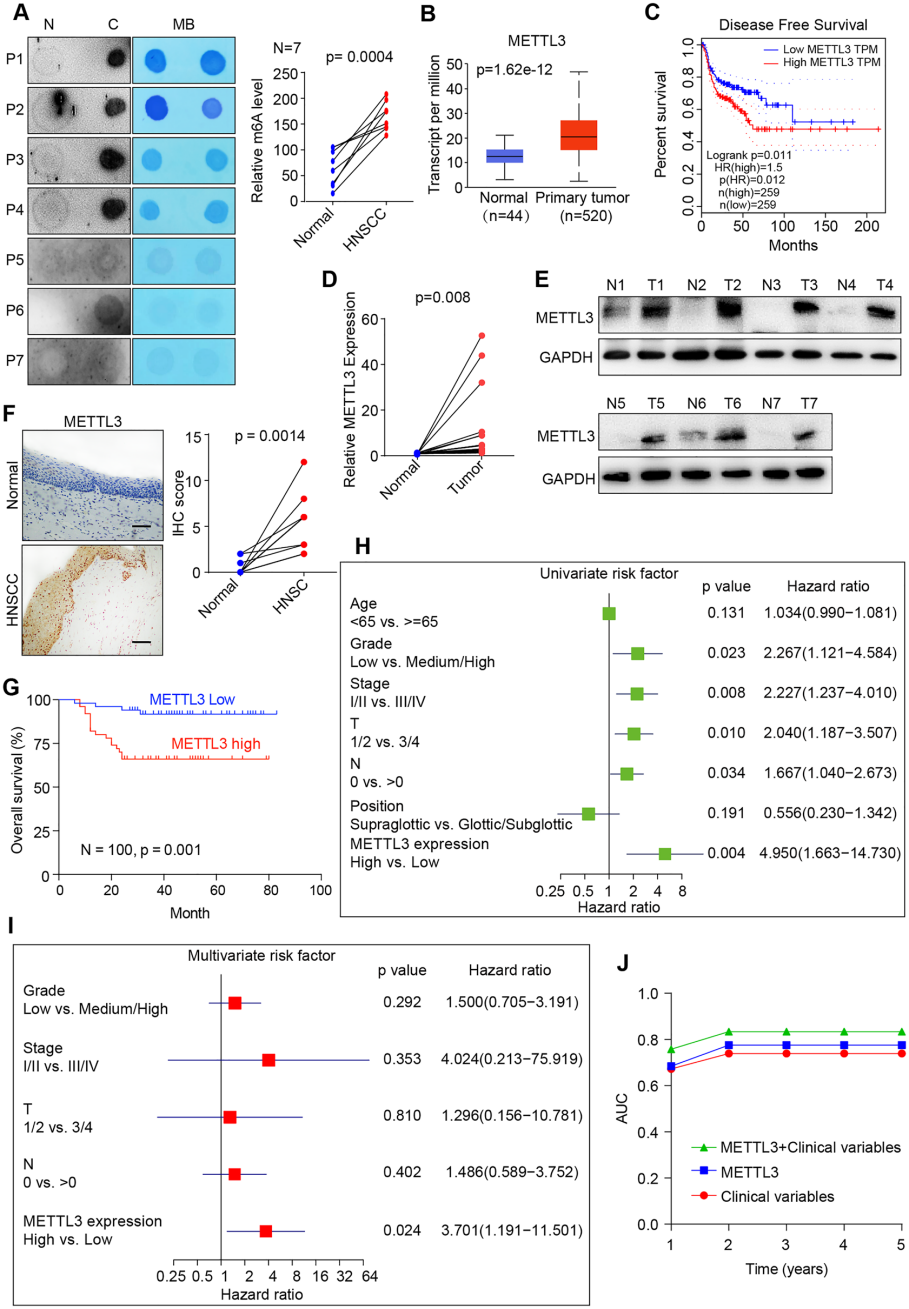
METTL3 high expression is associated with poor prognosis of HNSCC patients. **A** Dot blot assay was conducted with mRNA extracted from HNSCC tissues and paired paracancerous normal tissues using an anti-m6A antibody, and MB (methylene blue) staining served as the loading control (representative images in left panel). The relative m6A contents on mRNA in HNSCC tissues and paired normal normal tissues were calculated (right panel, n = 7). **B** TCGA data showed that METTL3 expression was significantly upregulated in HNSCC (n = 520) than normal tissue (n = 44). **C** Disease-free survival (RFS) of HNSCC patients based on METTL3 expression obtained from GEPIA website (http://gepia.cancer-pku.cn/). **D** The levels of METTL3 expression in HNSCC and paired normal tissues were measured by qRT-PCR (n = 10). **E** METTL3 protein levels were measured in HNSCC tissues and paired normal tissues by western blotting (n = 7). **F** METTL3 expression was significantly upregulated in HNSCC compared with the paired paracancerous normal tissue by IHC staining (n = 5, scale bars = 100 μm). **G** Kaplan–Meier OS analysis of HNSCC patients based on METTL3 expression measured by IHC of tissue microarray (n = 100). **H** Univariate Cox regression analysis was conducted in HNSCC patients (n = 100). All bars correspond to 95% confidence intervals. **I** Multivariate Cox regression analysis was conducted in HNSCC patients (n = 100). **J** The time-dependent receiver operating characteristic (ROC) analysis for the clinical risk score (TNM stage), the METTL3 risk score, and the combined METTL3 and clinical risk scores in HNSCC cohort

To investigate the role of m6A modification in HNSCC, we first examined the RNA m6A levels in 7 HNSCC tissues and paracancerous normal tissues via dot blot assay, and the results showed that RNA m6A levels were significantly higher in HNSCC tissues (Fig. 1A). Since m6A modification are regulated by its writers and erasers, and then we analyzed the expression of the main writers and erasers using the Cancer Genome Atlas (TCGA) data, and found the main m6A writers (METTL3, METTL14, WTAP, RBM15) and erasers (FTO, ALKBH5) were significantly increased in HNSCC tissues than normal tissues (Fig. 1B, Additional file 2: Figure S1A). However, high expression of METTL3 correlates with worse disease-free survival (DFS), while there was no correlation between the expression of other writers/erasers and prognosis of HNSCC patients (Fig. 1C, Additional file 1: Figure S1B). Further, we confirmed that METTL3 mRNA and protein expression were significantly higher in HNSCC tissues compared with paired normal tissues, respectively (Fig. 1D, E). In addition, these results were also confirmed by immunohistochemistry (IHC) staining, showing the expression of METTL3 was significantly increased in HNSCC tissues compared with that in matched normal tissues (Fig. 1F). Besides, high METTL3 expression in cancerous tissues was significant correlated with advanced T stage and poor tumor differentiation among HNSCC patients (Additional file 1: Table S1), and HNSCC patients with high expression of METTL3 had worse overall survival using our tissue microarray (TMA) (n = 100, p = 0.001; Fig. 1G, Additional file 3: Figure S2A). Simultaneously, univariate Cox regression analysis revealed that tumor grade, clinical stage, TNM stage, and METTL3 expression were substantially associated with the overall survival of HNSCC patients (Fig. 1H), and multivariate Cox regression analysis showed that only METTL3 expression was an independent prognostic factor for HNSCC patients (Fig. 1I). Meanwhile, we further analyzed the time-dependent receiver operating characteristic (ROC) curve to assess the predictive ability of METTL3 expression for HNSCC prognosis. When combined METTL3 risk score and clinical risk score, the area under the curve (AUC) is the highest, meaning that the combination of clinical risk score (TNM stage) and METTL3 risk score contributed much more than either one alone in these HNSCC patients (Fig. 1J). Taken together, these results revealed that m6A modification and METTL3 expression were higher in HNSCC and METTL3 may be an independent prognostic marker for HNSCC patients.

### METTL3 promotes HNSCC cell proliferation, migration, and invasion

**Fig. 2:**
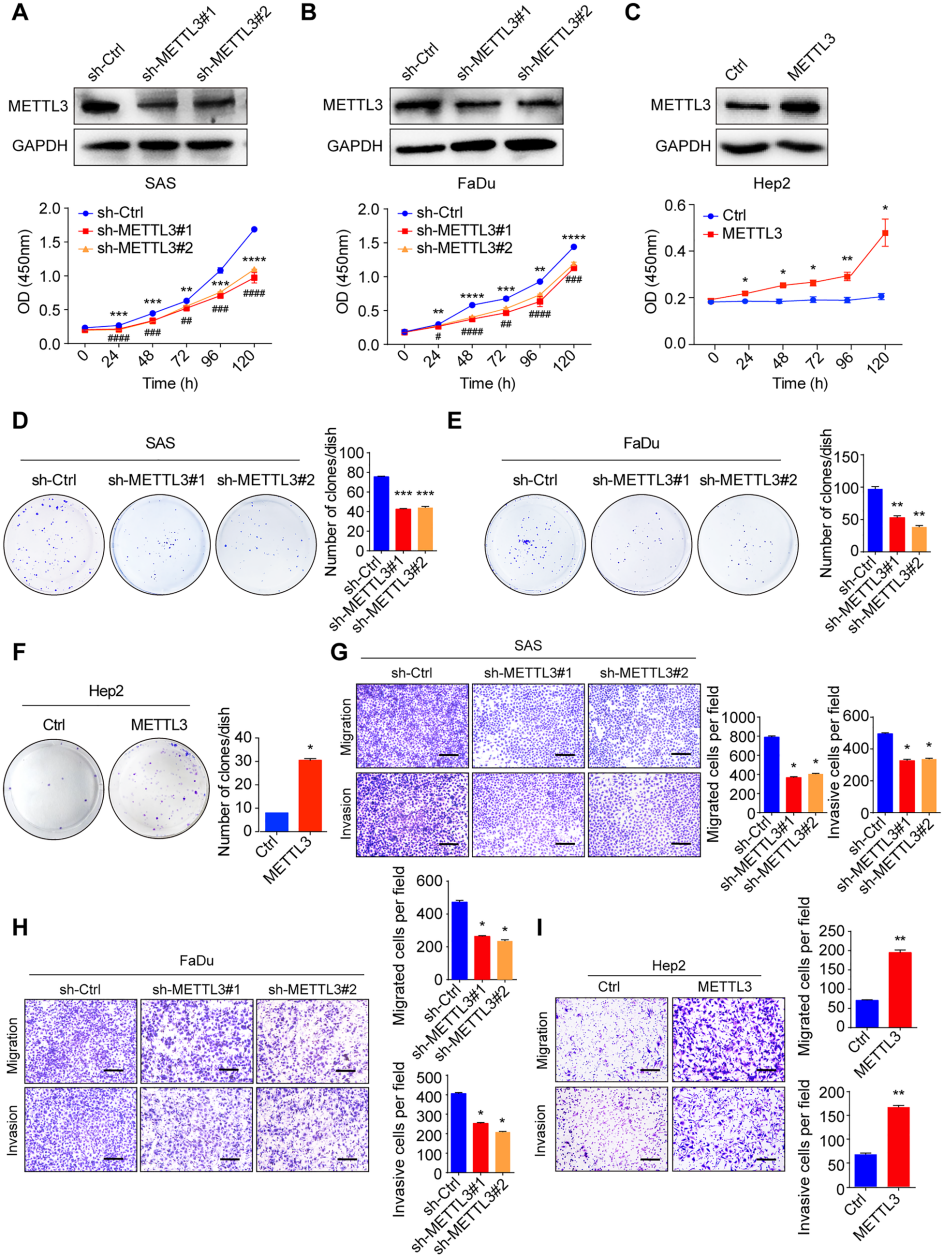
METTL3 promotes HNSCC cell proliferation, migration, and invasion. **A** The protein levels of METTL3 in SAS cells with METTL3 knockdown were measured by western blotting (upper panel), and cell proliferation ability in METTL3 knockdown and their corresponding control SAS cells were measured by a CCK8 assay (bottom panel). **B** The protein levels of METTL3 in FaDu cells with METTL3 knockdown were measured by western blotting (upper panel), and cell proliferation ability in METTL3 knockdown or control FaDu cells were measured by a CCK8 assay (bottom panel). **C** The protein levels of METTL3 in Hep2 cells with METTL3 overexpression were measured by western blotting (upper panel), and cell proliferation ability in METTL3 knockdown or control Hep2 cells were measured by a CCK8 assay (bottom panel). **D**, **E** knockdown of METTL3 inhibits HNSCC cell proliferation by colony formation assay (left panel). Quantification of the colony formation assay results (right panel). **F** METTL3 overexpression promotes HNSCC cell proliferation by colony formation assay (left panel). Quantification of the colony formation assay results (right panel). **G**, **H** knockdown of METTL3 inhibits HNSCC cell migration and invasion by transwell assays. Representative images (scale bars = 100 μm, left panel) and quantification (right panel) of the cell migration and invasion assay results were shown. **I** METTL3 overexpression promotes HNSCC cell migration and invasion by transwell assays. Representative images (scale bars = 100 μm, left panel) and quantification (right panel) of the cell migration and invasion assay results were shown. The data are the means ± SD of three independent experiments. */# p < 0.05; **/## p < 0.01; ***/### p < 0.001; ****/#### p < 0.0001

To investigate the function of METTL3 in HNSCC, we first detected its expression in five different HNSCC cell lines via qPCR and western blot (Additional file 3: figure S2B, C). The results showed that METTL3 was expressed relatively higher in SAS, FaDu, and Tu686, while lower in Hep2 and Tu212 cell lines. We then established the stable METTL3 knockdown cells (SAS and FaDu), and METTL3-overexpressing cells (Hep2) via METTL3 shRNA or overexpression lentivirus plasmid, respectively (Fig. 2A–C, Additional file 3: Figure S2D). Further, we conducted a series of functional studies, and the result of CCK8 assay showed that down-regulation of METTL3 significantly decreased cell proliferation, while up-regulation of METTL3 showed an opposite effect (Fig. 2A–C). The colony formation assay was also confirmed that down-regulation of METTL3 significantly decreased, while up-regulation of METTL3 increased the number of colonies (Fig. 2D–F). To determine the roles of METTL3 in HNSCC metastasis, we also performed migration and invasion assays, and the results revealed that knockdown of METTL3 suppressed the migration and invasion of SAS and FaDu cells, however, overexpression of METTL3 dramatically promoted these effects in Hep2 cells (Fig. 2G–I).

### METTL3 mediates the m6A modification of CDC25B in HNSCC

**Fig. 3:**
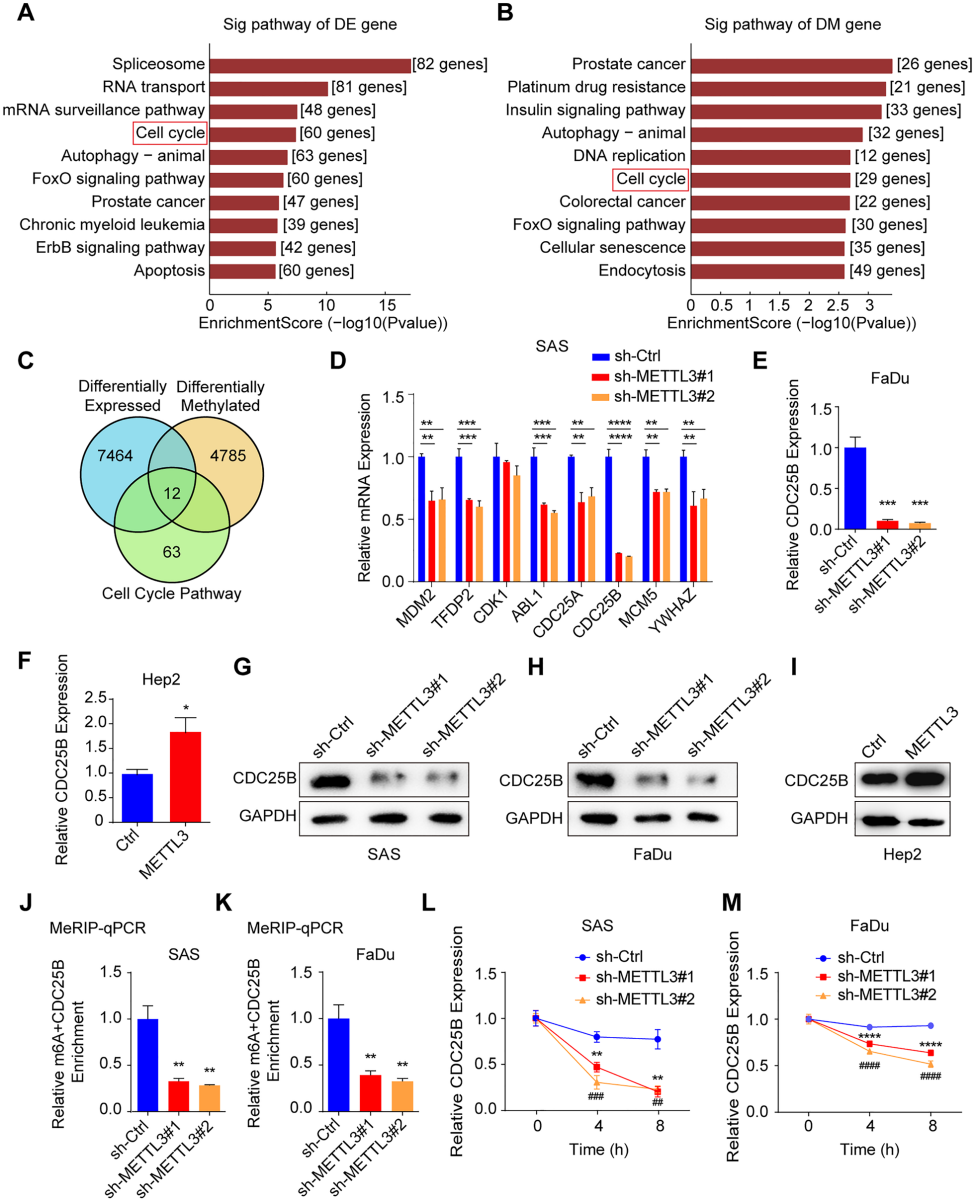
METTL3 mediates the m6A modification on CDC25B mRNA in HNSCC. **A** m6A-mRNA epitranscriptomic microarray showed signal pathways in which most differentially expressed gene enriched in METTL3 knockdown cells (SAS), DE represents differentially expressed. **B** m6A-mRNA epitranscriptomic microarray showed signal pathways in which most differentially methylated genes enriched in METTL3 knockdown cells (SAS), DM represents differentially methylated. **C** m6A-mRNA epitranscriptomic microarray showed an overlap of the total differentially expressed gene, total differentially methylated gene, and differentially expressed and methylated gene enriched in the cell cycle pathway. **D** Genes selected from the overlap were used for qRT-PCR in METTL3 knockdown and their corresponding control cells, and CDC25B was the most significantly downregulated gene upon knockdown of METTL3. **E**, **F** CDC25B mRNA expression was confirmed by qRT-PCR in METTL3 knockdown (FaDu) and METTL3 overexpression (Hep2) cells. **G** CDC25B protein level was measured by western blot assay in METTL3 knockdown SAS cells. **H** CDC25B protein level was measured by western blot assay in METTL3 knockdown FaDu cells. **I** CDC25B protein level was measured by western blot assay in METTL3 overexpressed Hep2 cells. **J** MeRIP-qPCR was conducted to detect the m6A level of CDC25B mRNA in METTL3 knockdown (SAS) cells. **K** MeRIP-qPCR was conducted to detect the m6A level of CDC25B mRNA in METTL3 knockdown (FaDu) cells. **L**, **M** The levels of CDC25B expression in METTL3 knockdown and their corresponding control cells treated with actinomycin D (5 μg/mL) at the indicated time points were detected by qRT-PCR. The data are the means ± SD of three independent experiments. */# p < 0.05; **/## p < 0.01; ***/### p < 0.001; ****/#### p < 0.0001

To explore the molecular mechanism by which METTL3 promotes HNSCC progression, we performed the m6A-mRNA epitranscriptomic microarray in METTL3 knockdown and the corresponding control HNSCC cells. The results showed that 7476 transcripts were significantly down-regulated (fold change > 2) and m6A peaks of 4797 transcripts exhibited significantly decreased abundance (fold change > 2) on METTL3 knockdown (Additional file 4: Figure S3A, B). Differentially expressed genes in mRNA expression and m6A-modified peaks were enriched in many signal pathways, and the cell cycle pathway is overlapped (Fig. 3A, B). Furthermore, we found 12 cell cycle-related genes were overlapped (Fig. 3C). Then, the mRNA levels of 8 genes (fold change > 2) from the 12 candidates were verified in the METTL3 knockdown SAS cells. It showed that the expression of CDC25B was the most significantly down-regulated (Fig. 3D), which was also confirmed in FaDu cells with METTL3 deficiency (Fig. 3E). Consistently, CDC25B was upregulated in Hep2 cells with METTL3 overexpression (Fig. 3F). Meanwhile, TCGA data also showed a positive correlation between METTL3 and CDC25B (Additional file 4: Figure S3C). Further, CDC25B protein levels were also positively regulated by METTL3 in different HNSCC cell lines (Fig. 3G–I). In addition, the enrichment of m6A modification on CDC25B mRNA was significantly decreased in METTL3 knockdown cells compared with the corresponding control cells using MeRIP-qPCR assay (Fig. 3J, K). Furthermore, when cells were treated with transcription inhibitor actinomycin D, CDC25B mRNA was shown to be less stable on METTL3 knockdown (Fig. 3L, M). These data suggest that METTL3-mediated CDC25B m6A modification and enhanced its mRNA stability regulates its expression in HNSCC.

### CDC25B promotes HNSCC cell proliferation, migration, invasion and cell cycle progression

**Fig. 4:**
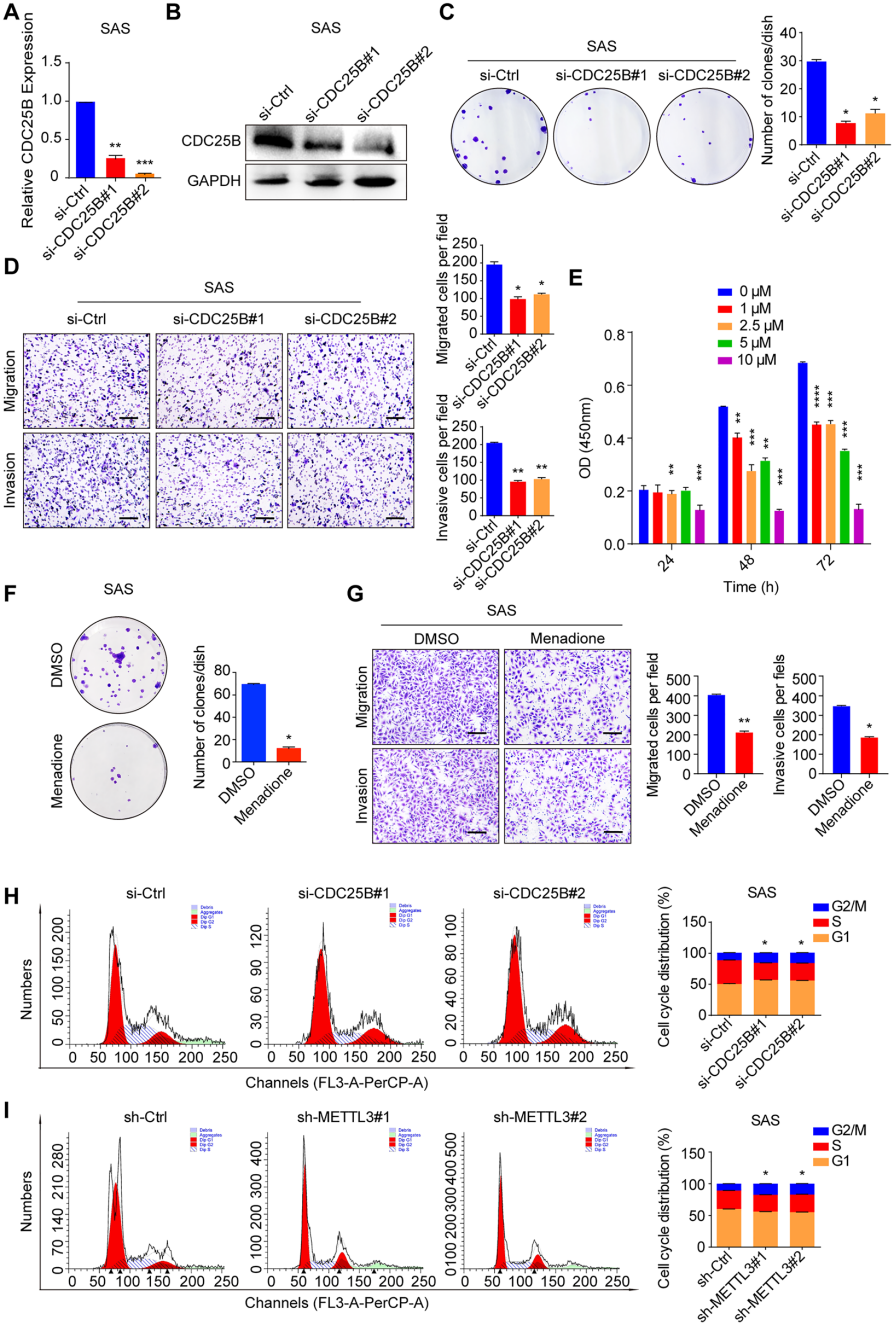
CDC25B promotes SAS cell proliferation, migration, invasion, and cell cycle progression. **A** The qRT-PCR was conducted to confirm CDC25B knockdown efficiency at mRNA level. **B** Western blot assay was conducted to confirm CDC25B knockdown efficiency at protein level. **C** Knockdown of CDC25B inhibited cell proliferation in colony formation assay (left panel); quantification results of colony formation (right panel). **D** CDC25B knockdown inhibited cell migration and invasion by transwell assays. Representative images (scale bars = 100 μm, left panel) and quantification (right panel) of the cell migration and invasion assay results were shown. **E** CCK8 assay was conducted on SAS cell after different concentrations of CDC25B inhibitor (menadione) treatment at indicated time. **F** CDC25B inhibitor (menadione) concentration of 5 μM was used for constant inhibition in colony formation assay and showed that menadione can inhibit cell proliferation (upper panel); quantification results of colony formation (bottom panel). **G** SAS cells were treated with 5 μM menadione for 24 h and used for transwell assays, showing that menadione can inhibit cell migration and invasion. Representative images (scale bars = 100 μm, left panel) and quantification (right panel) of the cell migration and invasion assay results were shown. Cell cycle G2/M arrest was observed in CDC25B knockdown cells (**H**) and METTL3 knockdown cells (**I**). The data are the means ± SD of three independent experiments. * p < 0.05; ** p < 0.01; *** p < 0.001; **** p < 0.0001

To further investigate the role of CDC25B in HNSCC, we first designed 2 different specific siRNAs targeting CDC25B and confirmed their knockdown efficiency via qPCR and western blot assay (Fig. 4A, B, Additional file 5: Figure S4A, B). Knockdown of CDC25B significantly suppressed the colony formation, cell migration and invasion (Fig. 4C, D, Additional file 5: Figure S4C, D). Furthermore, it showed that menadione, a special inhibitor of CDC25B, could significantly inhibit HNSCC cell growth with an increase of treatment times and doses (Fig. 4E, Additional file 5: Figure S4E). It also indicated that there was no obvious cytotoxicity with the treatment of 5 μM menadione for 24 h (Fig. 4E, Additional file 5: Figure S4E). However, the ability of colony formation, cell migration and invasion were dramatically decreased in the cells treated with 5 μM menadione for 24 h (Fig. 4F, G, Additional file 5: Figure S4F, G). Considering CDC25B is a cell cycle kinase, cell cycle in HNSCC cells was detected using flow cytometry assay. We found G2/M arrest upon CDC25B knockdown or treated with menadione, as well as METTL3 knockdown (Fig. 4H, I, Additional file 5: Figure S4H, I, Additional file 6: Figure S5A, B). Thus, our data suggested that CDC25B could regulate HNSCC progression.

### METTL3 promotes HNSCC progression via modulating CDC25B m6A modification

**Fig. 5:**
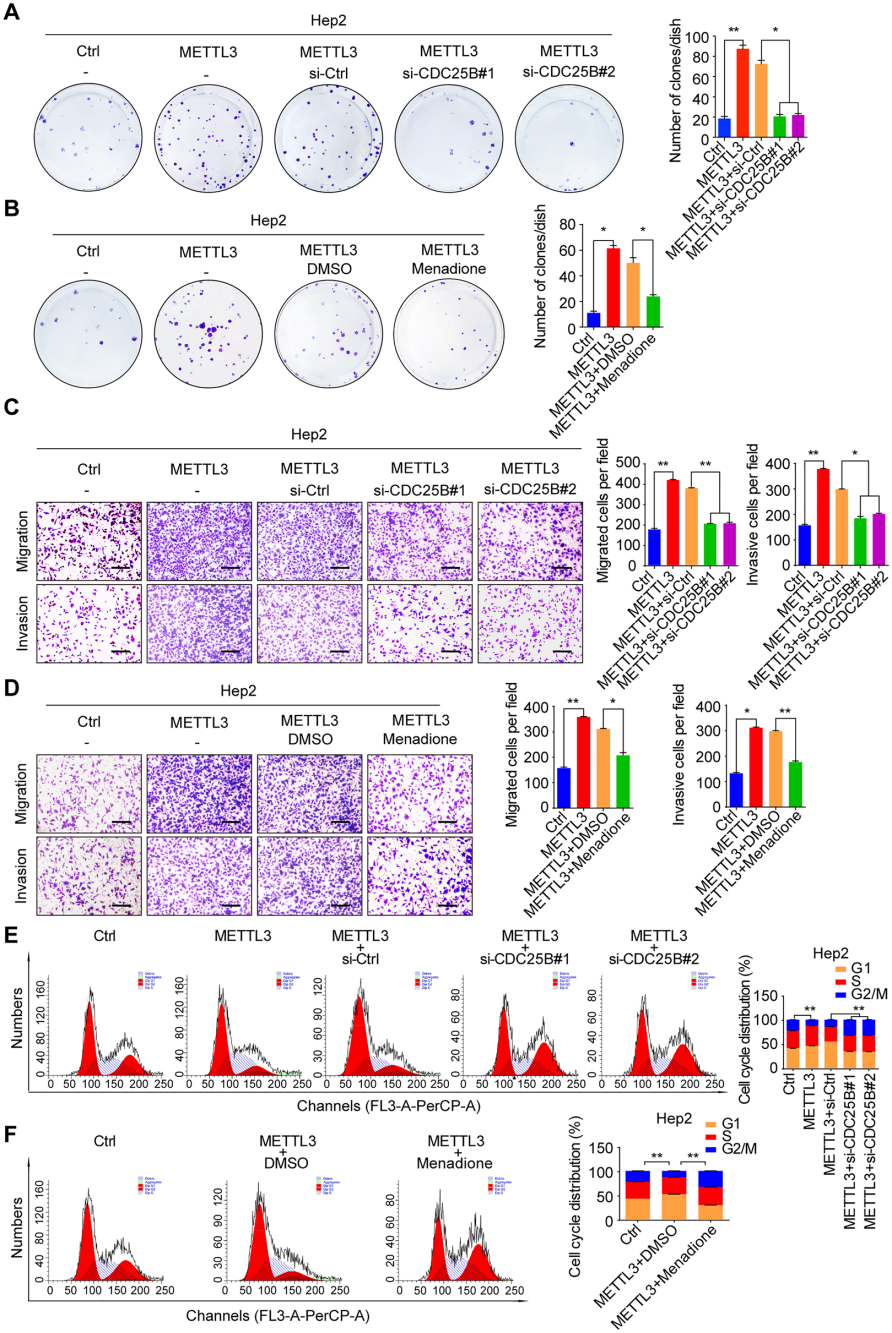
METTL3 accelerates HNSCC malignant progression by upregulating CDC25B. **A** Colony formation assay of METTL3 overexpressing Hep2 cells transfected with CDC25B siRNAs and the corresponding control (left panel), numbers of the colony formation were calculated (right panel). **B** Colony formation assay of METTL3 overexpressing Hep2 cells treated with 5 μM menadione or DMSO for 24 h (left panel), numbers of the colony formation were calculated (right panel). **C** Transwell assay of METTL3 overexpressing Hep2 cells transfected with CDC25B siRNAs and the corresponding control (left panel) to evaluate cell migration and invasion ability (scale bars = 100 μm, left panel), numbers of cells migrated and invaded were calculated (right panel). **D** Transwell assay of METTL3 overexpressing Hep2 cells treated with 5 μM menadione or DMOS for 24 h and the corresponding control (scale bars = 100 μm, left panel), numbers of cells migrated and invaded were calculated (right panel). **E** Cell cycle assay of METTL3 overexpressing Hep2 cells transfected with CDC25B siRNAs and the corresponding control (left panel), percentage of cell cycle phase was calculated (right panel). **F** Cell cycle assay of METTL3 overexpressing Hep2 cells treated with 5 μM menadione or DMSO for 24 h and the corresponding control (left panel), percentage of cell cycle phase was calculated (right panel). The data are the means ± SD of three independent experiments. * p < 0.05; ** p < 0.01

We further confirm the functional role of METTL3-mediated m6A modification of CDC25B in HNSCC. As expected, the expression of CDC25B was knocked down in METTL3-overexpressing Hep2 cells using its specific siRNAs, which markedly suppressed METTL3-induced HNSCC cell proliferation (Fig. 5A). Similarly, it also showed the similar phenotype using 5 μM menadione to inhibit CDC25B activity (Fig. 5B). Meanwhile, the HNSCC cell migration and invasion ability were significantly increased upon METTL3 overexpression but were reversely inhibited upon knockdown of CDC25B expression or inhibition its activity with menadione (Fig. 5C, D). In addition, upregulation of METTL3 promoted cell cycle progression and G2/M proportion was decreased, but knockdown of CDC25B expression or inhibition of its activity with menadione, the G2/M transition was arrested (Fig. 5E, F). Thus, the data suggest that METTL3 promotes HNSCC malignant progression through the upregulation of CDC25B.

### METTL3 accelerates tumor growth and angiogenesis in vivo

**Fig. 6:**
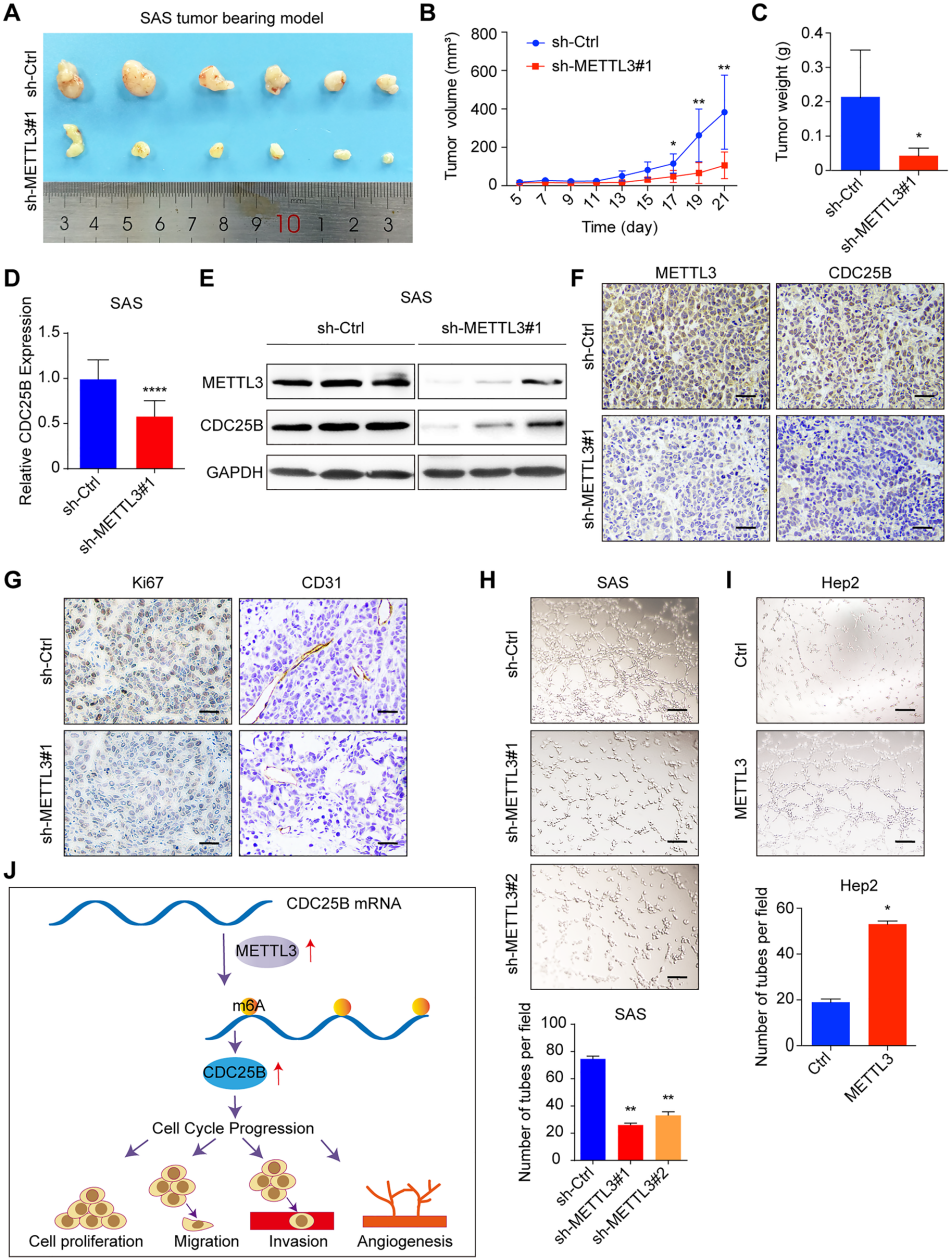
Overexpression of METTL3 promotes tumor growth and angiogenesis. **A** Knockdown of METTL3 significantly inhibits subcutaneous tumor growth in nude mice (n = 6). **B** The tumor volume curve was conducted by measuring every other day. **C** The mice were executed and tumors were extracted and weighed after 21 days. **D** CDC25B mRNA level in extracted tumor tissues from METTL3 knockdown and corresponding control groups was measured by qRT-PCR. **E** METTL3 and CDC25B protein level in extracted tumors from METTL3 knockdown and corresponding control groups was measured by western blot assay. **F** METTL3 and CDC25B protein level was measured by IHC on extracted tumors from METTL3 knockdown and corresponding control groups (scale bars = 100 μm). **G** Ki67 and CD31 protein level was measured by IHC on extracted tumors from METTL3 knockdown and corresponding control groups (scale bars = 100 μm). **H** Tube formation assay was conducted using HUVEC cultured in medium from METTL3 knockdown SAS cells and their corresponding control cells (upper panel), and the number of tubes was calculated (bottom panel). **I** Tube formation assay was conducted using HUVEC cultured in medium from METTL3 overexpressing Hep2 cells and their corresponding control cell (upper panel), and the number of tubes was calculated (bottom panel; scale bars = 100 μm). **J** The graphic illustration of METTL3 mediated the m6A modification of CDC25B mRNA, promoting HNSCC cell cycle progression to lead to the cell proliferation, migration, invasion, and angiogenesis. The data are the means ± SD of three independent experiments. * p < 0.05; ** p < 0.01; **** p < 0.0001

To investigate the function of METTL3 in vivo, we also performed tumor xenograft in nude mice. It showed that knockdown of METTL3 significantly restrained tumor growth (Fig. 6A), as reflected by tumor volume and weight (Fig. 6B, C). As confirmed by qPCR and western blot, CDC25B expression was downregulated upon knockdown of METTL3 in tumor tissues (Fig. 6D, E). Consistently, IHC results also showed that CDC25B staining was also obviously decreased in tumor xenograft with deficiency of METTL3 (Fig. 6F). In addition, IHC staining with Ki67 confirmed that knockdown of METTL3 suppressed cell proliferation as its expression decreased in the knockdown group (Fig. 6G). We also found METTL3 was positively correlated with the proliferation markers (Ki67 and PCNA) in HNSCC TCGA data (Additional file 6: figure S5C, D). Furthermore, we found that tumors derived from vector control cells exhibited more blood vessels (Fig. 6A) and it also showed a positively correlation between METTL3 and VEGFA in TCGA database of HNSCC (Additional file 6: Figure S5E). IHC staining of CD31, a marker of angiogenesis, also showed a significant decreased in tumor tissues of the METTL3 knockdown group compared with that in the control group (Fig. 6G). To further study the function of METTL3 in HNSCC angiogenesis, human umbilical vein endothelial cell (HUVEC) tube formation was investigated in vitro. The HUVEC tube formation was significantly impaired by conditioned medium from SAS cells with knockdown of METTL3 compared with conditioned medium from the vector controls (Fig. 6H); Correspondingly, tube formation was significantly increased by conditioned medium from Hep2 cells with METTL3 overexpression (Fig. 6I). Collectively, these results suggest that METTL3/CDC25B promotes HNSCC tumor growth and angiogenesis.

## Discussion

In the present study, we demonstrated that m6A modification and METTL3 levels are increased in HNSCC, and METTL3 might be an independent prognostic factor for HNSCC patients. Mechanistically, METTL3 acts as an oncogene to promote cell proliferation, migration, invasion, and angiogenesis in HNSCC through the m6A-mediated upregulation of CDC25B (Fig. 6J).

Epigenetic modifications, especially the abnormal genome methylations have been widely reported in the malignant progression of HNSCC. For example, the tumor suppressor NDRG1 and NDRG2 present highly methylated and low protein expression status in laryngeal cancer, suggesting that abnormal methylation on the NDRG1/2 promoter region may be an early event in laryngeal cancer initiation [40]. In oral squamous cell carcinoma that happened in an Asian population, hypomethylation of Alu, a short interspersed element, decreased as the tumor progresses [41]. Some studies also showed that widely hypomethylation occur in tongue squamous cell carcinoma [42]. It also reported that hypermethylation of the promoter region of the tumor suppressor PTEN could be seen in oral squamous cell carcinoma and nasopharyngeal carcinoma [43–45].

RNA epigenetic modification is the chemical basis for RNA regulation, and more than 170 modifications have been identified [46, 47]. With some related enzymes, RNA could have many epigenetic modifications like N6-Methyladenosine (m6A), pseudouridine (Ψ), 5-methylcytosine 57 (m5C), 5-hydroxymethylcytosine (5hmC), 7-methylguanosine(m7G), and N1-methyladenosine (m1A), which can affect the RNA stability, translation efficiency and others [12, 48, 49]. M6A is the most prevalent in mRNA and non-coding RNA, and recent studies have revealed that m6A and its related regulators play a critical role in various cancers, including cervical cancer [50], breast cancer [51], pancreatic cancer [52], esophageal cancer [53], and prostate cancer [54]. The m6A modification is mainly regulated by m6A methyltransferases (writers), demethylases (erasers), and specific RNA-binding proteins (readers) [14]. Lots of studies showed that altered the expression of m6A regulators could increase the occurrence and development of digestive system tumour, and modulation of m6A modification could be the potential therapy for tumour. For example, tumour cells may become more sensitive to chemotherapy, radiotherapy and immunotherapy upon overexpression or knockout of the m6A-related regulators [55]. Here, we indicate that in HNSCC, upregulation of the m6A methyltransferase METTL3 increases m6A level and is associated with worse clinical prognosis for HNSCC patients. In addition, high expression of METTL3 was associated with high T stage and low differentiation. Further, in vitro and in vivo studies indicated that METTL3 promotes HNSCC tumor growth, as well as cell proliferation, migration, invasion, cell cycle progression, and angiogenesis. Consequently, our data suggested that METTL3 may act as an independent prognostic factor and therapeutic target for HNSCC patients.

From m6A-mRNA epitranscriptomic microarray, we found that CDC25B was the pivotal downstream molecular of METTL3 in HNSCC. CDC25B is a cell cycle related enzyme that is critical for G2/M transition. Studies have showed that highly expression of CDC25B in many types of cancer like esophageal squamous carcinoma [56], colorectal cancer [57], non-small cell lung cancer [58], endometrial carcinoma [59], pancreatic ductal adenocarcinoma [60], and breast cancer [61]. However, the function of CDC25B have not been reported in HNSCC. CDC25B can dephosphorylate and activate AMP-activated protein kinase signaling (AMPL) by inhibiting protein phosphatase 2A (PPA2) [62]. Our data showed that in HNSCC, CDC25B was positively regulated by METTL3 by catalyzing the m6A modification on its mRNA, leading to increased cell proliferation, migration, invasion, and cell cycle progression.

## Conclusion

our findings indicate that METTL3 acts as an oncogene in HNSCC by regulating m6A modification of CDC25B, a cell cycle protein. Moreover, METTL3 is expressed significantly higher in HNSCC and is associated with a poor prognosis. Consequently, METTL3 may provide a promising future in HNSCC diagnosis and treatment.

## Supplementary Information

**Additional file 1: Table S1**. Relationship between METTL3 expression and clinicopathological features in HNSCC patients. **Table S2**. Primer sequences and siRNA sequences. **Table S3**. Antibodies for western blot and IHC

**Additional file 2: Figure S1**. Expression differences and prognosis relation of m6A related proteins in HNSCC. (A) Expression of 6 m6A related proteins (METTL14, WTAP, RBM15, KIAA1429, FTO, ALKBH5) in normal tissues and HNSCC tissues using TCGA data. (B) Disease-free survival (RFS) of HNSCC patients based on the expression of METTL14, WTAP, RBM15, KIAA1429, FTO, ALKBH5 obtained from GEPIA website (http://gepia.cancer-pku.cn/). * p<0.05; ** p<0.01; *** p<0.001.

**Additional file 3: Figure S2**. METTL3 protein and mRNA level in HNSCC and HNSCC cell lines. (A) IHC of METTL3 in HNSCC tissue microarray, evaluation standard was tagged (scale bars=100μm). (B) QRT-PCR assay was used to evaluate METTL3 mRNA level in 5 HNSCC cell lines (SAS, FaDu, Hep2, Tu212, Tu686). (C) Western blotting assay was used to evaluate METTL3 protein level in 5 different HNSCC cell lines. (D) QRT-PCR assay was used to evaluate the METTL3 knockdown and overexpression efficiency in 3 different HNSCC cell lines (SAS, FaDu, Hep2). The data are the means±SD of three independent experiments. * p<0.05; ** p<0.01.

**Additional file 4: Figure S3**. METTL3 promotes CDC25B expression in HNSCC. (A-B) Differentially expressed genes in mRNA expression and methylation were enriched. (C) METTL3 expression has a positive correlation with CDC25B expression in HNSCC (GEPIA website, http://gepia.cancer-pku.cn/). The data are the means±SD of three independent experiments. ** p<0.01.

**Additional file 5: Figure S4**. CDC25B promotes cell proliferation, migration, invasion, and cell cycle progression in FaDu cells. (A) The qRT-PCR was conducted to confirm CDC25B knockdown efficiency at mRNA level. (B) Western blot assay was conducted to confirm CDC25B knockdown efficiency at protein level. (C) Knockdown of CDC25B inhibited cell proliferation in colony formation assay (left panel); quantification results of colony formation (right panel). (D) CDC25B knockdown inhibited cell migration and invasion by transwell assays. Representative images (scale bars=100μm, left panel) and quantification (right panel) of the cell migration and invasion assay results were shown. (E) CCK8 assay was conducted on FaDu cell after different concentrations of CDC25B inhibitor (menadione) treatment at indicated time. (F) CDC25B inhibitor (menadione) concentration of 5μM was used for constant inhibition in colony formation assay and showed that menadione can inhibit cell proliferation (left panel); quantification results of colony formation (right panel). (G) FaDu cells were treated with 5μM menadione for 24h and used for transwell assays, showing that menadione can inhibit cell migration and invasion. Representative images (scale bars=100μm, left panel) and quantification (right panel) of the cell migration and invasion assay results were shown. Cell cycle G2/M arrest was observed in CDC25B knockdown cells (H) and METTL3 knockdown cells (I). The data are the means±SD of three independent experiments. * p<0.05; ** p<0.01; *** p<0.001; **** p<0.0001.

**Additional file 6: Figure S5**. CDC25B inhibition results in G2/M arrest, and METTL3 expression has a positive correlation with MKI67, PCNA, and VEGFA. (A) Cell cycle assay of METTL3 knockdown SAS cells treated with 5μM menadione or DMSO for 24h and the corresponding control (left panel), percentage of cell cycle phase were calculated (right panel). (B) Cell cycle assay of METTL3 knockdown FaDu cells treated with 5μM menadione or DMSO for 24h and the corresponding control (left panel), percentage of cell cycle phase were calculated (right panel). (C, D, E) METTL3 expression has a positive correlation with MKI67, PCNA and VEGFA in HNSCC (GEPIA website).

## Data Availability

Contact the author if need the datasets generated during and/or analysed during the current study.

## Abbreviations

3'UTR: 3'Untranslated region
5hmC: 5-Hydroxymethylcytosine
ALKBH5: Alpha-ketoglutarate-dependent dioxygenase alkB homolog 5
Alu: Alumine
AML: Acute myeloid leukemia
AMP: Adenylyl imidodiphosphate
ANOVA: Analysis of variance
CCK8: Cell Counting Kit-8
CD31: Cluster of differentiation 31
CDC25B: Cell division cycle 25 B
CDK1: Cyclin-dependent kinases 1
DFS: Disease-free survival
DMEM: Dulbecco's Modified Eagle Medium
DMSO: Dimethyl sulfoxide
FBS: Fetal bovine serum
FTO: Alpha-ketoglutarate-dependent dioxygenase
GO analysis: Gene ontology analysis
HNSCC: Head and neck squamous cell carcinoma
HPV: Human papillomavirus
HUVEC: Human umbilical vein endothelial cell
IgG: Immunoglobulin G
IHC: Immunohistochemical
MKI67: Marker of proliferation Ki-67
m1A: N1-methyladenosine
m5C: 5-Methylcytosine 57
m6A: N6-methyladenosine
m7G: 7-Methylguanosine
MeRIP-qPCR: Methyl-RNA immunoprecipitation and quantitative polymerase chain reaction
METTL14: Methyltransferase Like 14
METTL3: Methyltransferase Like 3
mRNA: Messenger RNA
NDRG1/2: N-Myc Downstream Regulated 1/2
NSCLC: Non-small cell lung cancer
PBS: Phosphate buffered saline
PCNA: Proliferating cell nuclear antigen
PTEN: Phosphatase and tensin homolog
QRT-PCR: Quantitative real-time polymerase chain reaction
RBM15: RNA binding motif protein 15
ROC curve: Receiver operating characteristic curve
RPMI-1640: Roswell Park Memorial Institute 1640
shRNA: Short hairpin RNA
siRNA: Small interfering RNA
TCGA: The Cancer Genome Atlas
TMA: Tissue microarray
TNM stage: Tumour, node and metastasis stage
VEGFA: Vascular Endothelial Growth Factor A
WTAP: WT1 associated protein
