# Supplementary figures and images for: METTL3 modulates m6A modification of CDC25B and promotes head and neck squamous cell carcinoma malignant progression
Source: Exp Hematol Oncol. 2022 Mar 14;11:14. doi: 10.1186/s40164-022-00256-3 (PMC8919647; doi:10.1186/s40164-022-00256-3)

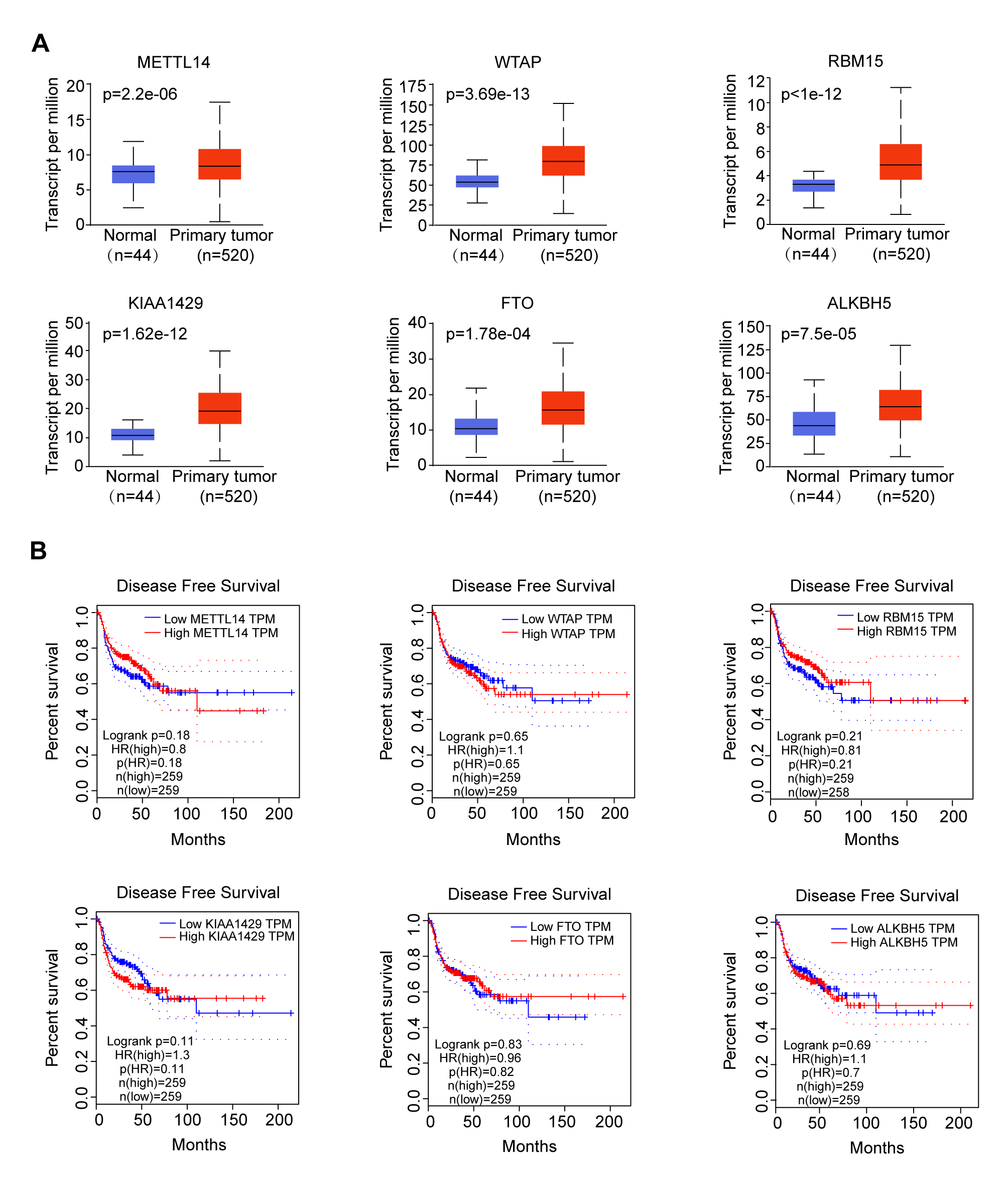

Supplement: Supplementary file 2 — Additional file 2: Figure S1. Expression differences and prognosis relation of m6A related proteins in HNSCC. (A) Expression of 6 m6A related proteins (METTL14, WTAP, RBM15, KIAA1429, FTO, ALKBH5) in normal tissues and HNSCC tissues using TCGA data. (B) Disease-free survival (RFS) of HNSCC patients based on the expression of METTL14, WTAP, RBM15, KIAA1429, FTO, ALKBH5 obtained from GEPIA website (http://gepia.cancer-pku.cn/). * p<0.05; ** p<0.01; *** p<0.001. [file 40164_2022_256_MOESM2_ESM.tif]

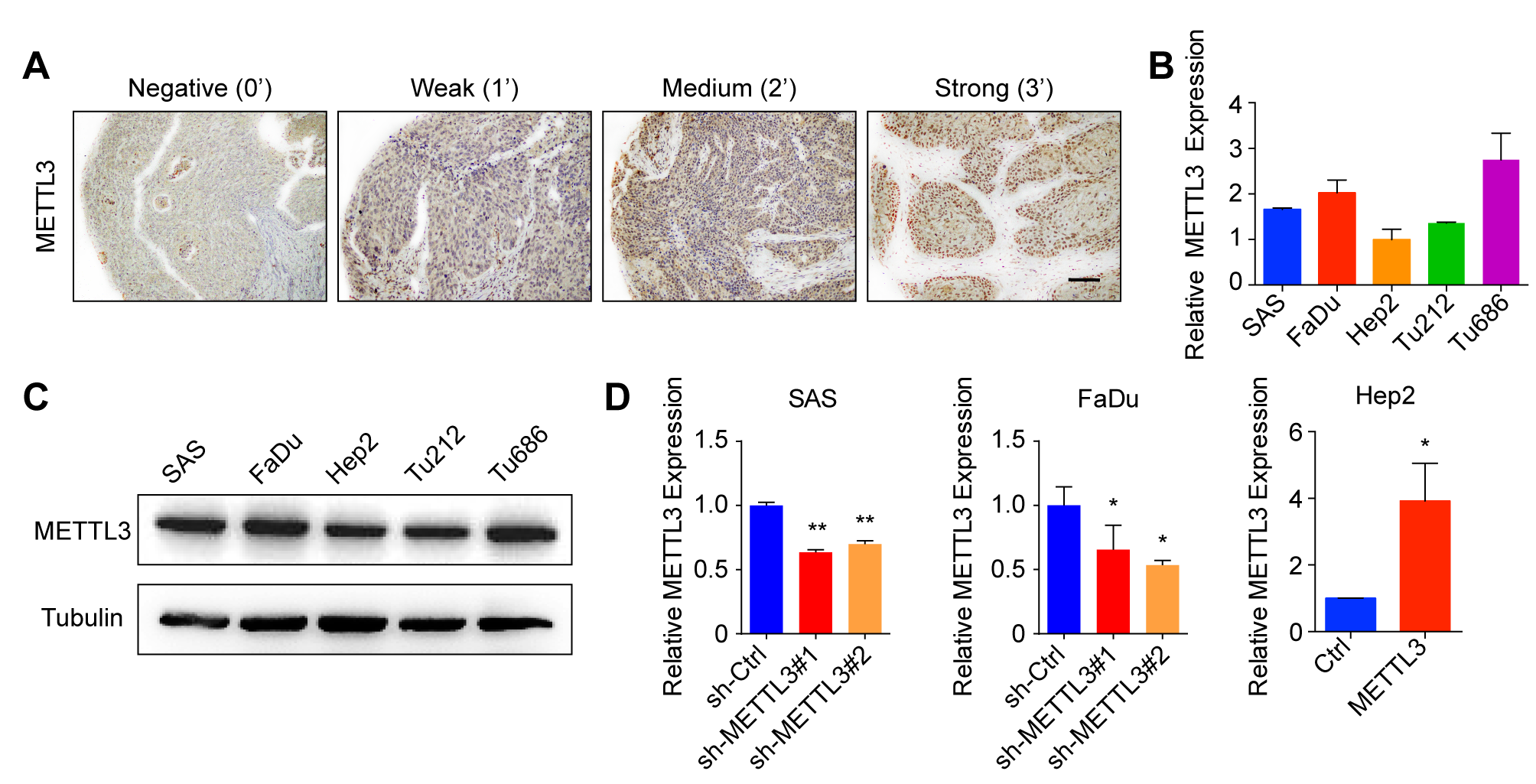

Supplement: Supplementary file 3 — Additional file 3: Figure S2. METTL3 protein and mRNA level in HNSCC and HNSCC cell lines. (A) IHC of METTL3 in HNSCC tissue microarray, evaluation standard was tagged (scale bars=100μm). (B) QRT-PCR assay was used to evaluate METTL3 mRNA level in 5 HNSCC cell lines (SAS, FaDu, Hep2, Tu212, Tu686). (C) Western blotting assay was used to evaluate METTL3 protein level in 5 different HNSCC cell lines. (D) QRT-PCR assay was used to evaluate the METTL3 knockdown and overexpression efficiency in 3 different HNSCC cell lines (SAS, FaDu, Hep2). The data are the means±SD of three independent experiments. * p<0.05; ** p<0.01. [file 40164_2022_256_MOESM3_ESM.tif]

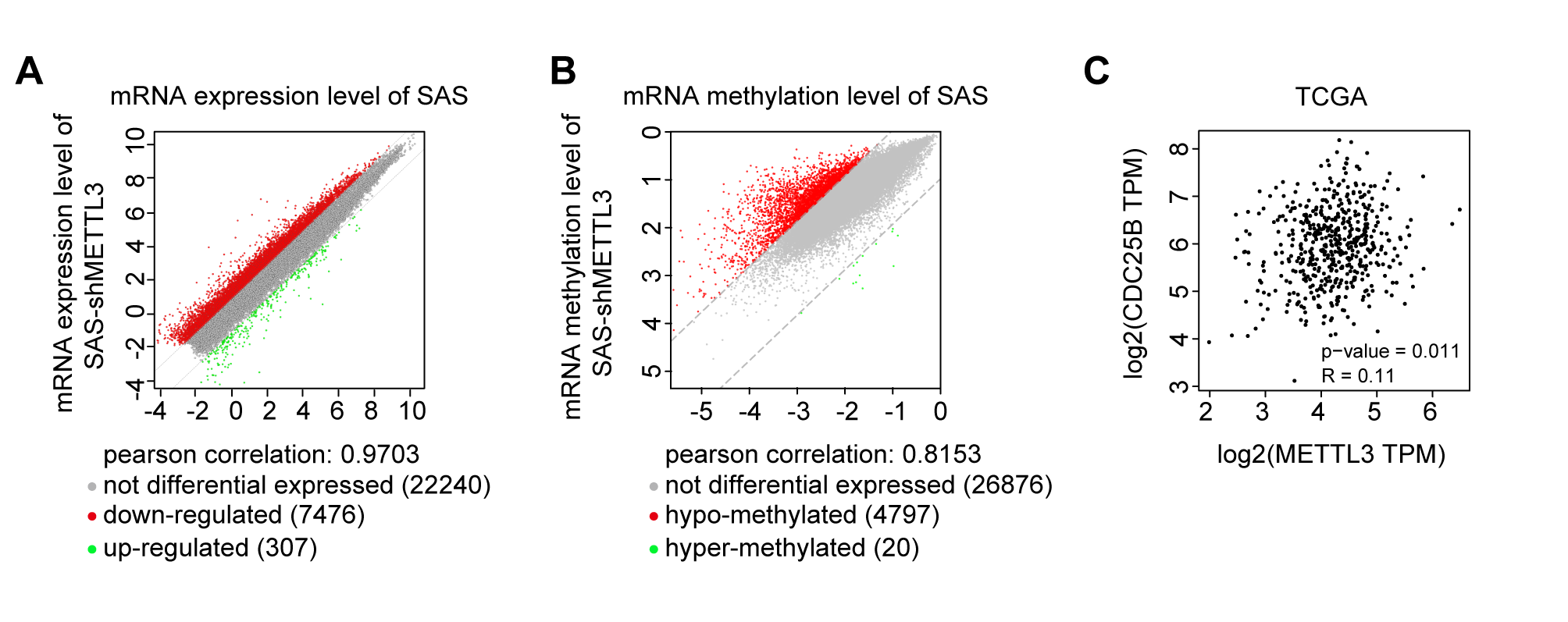

Supplement: Supplementary file 4 — Additional file 4: Figure S3. METTL3 promotes CDC25B expression in HNSCC. (A-B) Differentially expressed genes in mRNA expression and methylation were enriched. (C) METTL3 expression has a positive correlation with CDC25B expression in HNSCC (GEPIA website, http://gepia.cancer-pku.cn/). The data are the means±SD of three independent experiments. ** p<0.01. [file 40164_2022_256_MOESM4_ESM.tif]

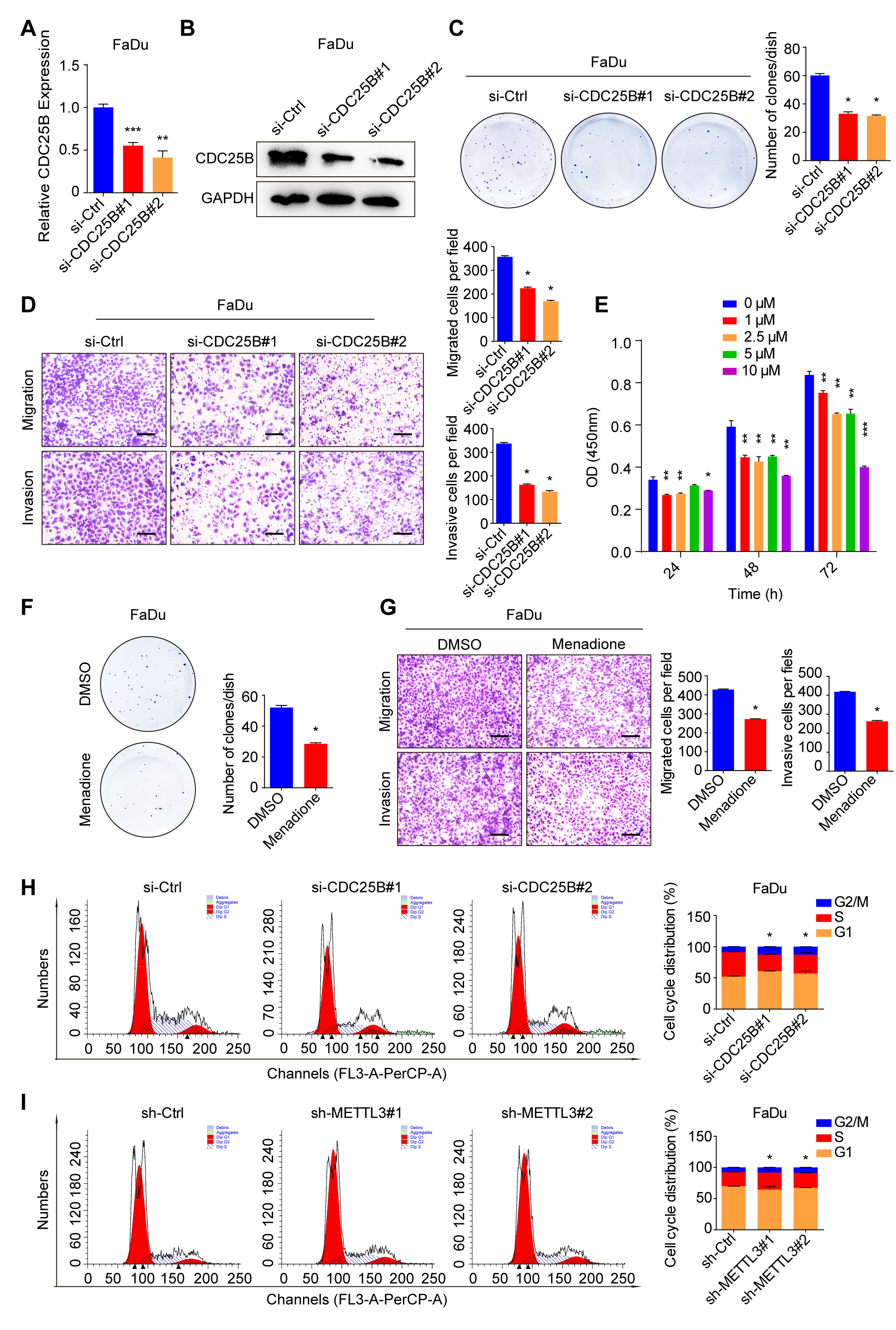

Supplement: Supplementary file 5 — Additional file 5: Figure S4. CDC25B promotes cell proliferation, migration, invasion, and cell cycle progression in FaDu cells. (A) The qRT-PCR was conducted to confirm CDC25B knockdown efficiency at mRNA level. (B) Western blot assay was conducted to confirm CDC25B knockdown efficiency at protein level. (C) Knockdown of CDC25B inhibited cell proliferation in colony formation assay (left panel); quantification results of colony formation (right panel). (D) CDC25B knockdown inhibited cell migration and invasion by transwell assays. Representative images (scale bars=100μm, left panel) and quantification (right panel) of the cell migration and invasion assay results were shown. (E) CCK8 assay was conducted on FaDu cell after different concentrations of CDC25B inhibitor (menadione) treatment at indicated time. (F) CDC25B inhibitor (menadione) concentration of 5μM was used for constant inhibition in colony formation assay and showed that menadione can inhibit cell proliferation (left panel); quantification results of colony formation (right panel). (G) FaDu cells were treated with 5μM menadione for 24h and used for transwell assays, showing that menadione can inhibit cell migration and invasion. Representative images (scale bars=100μm, left panel) and quantification (right panel) of the cell migration and invasion assay results were shown. Cell cycle G2/M arrest was observed in CDC25B knockdown cells (H) and METTL3 knockdown cells (I). The data are the means±SD of three independent experiments. * p<0.05; ** p<0.01; *** p<0.001; **** p<0.0001. [file 40164_2022_256_MOESM5_ESM.tif]

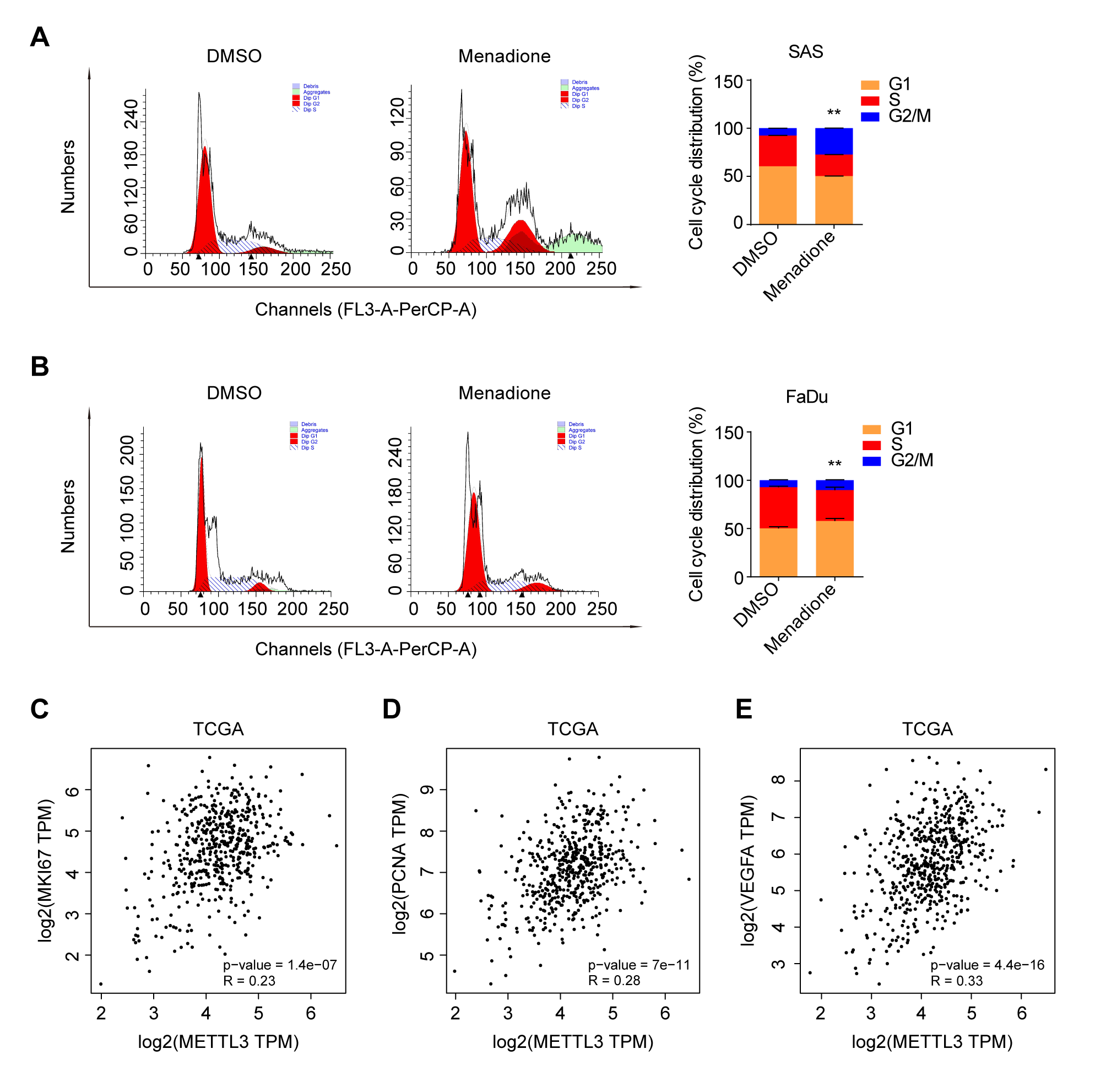

Supplement: Supplementary file 6 — Additional file 6: Figure S5. CDC25B inhibition results in G2/M arrest, and METTL3 expression has a positive correlation with MKI67, PCNA, and VEGFA. (A) Cell cycle assay of METTL3 knockdown SAS cells treated with 5μM menadione or DMSO for 24h and the corresponding control (left panel), percentage of cell cycle phase were calculated (right panel). (B) Cell cycle assay of METTL3 knockdown FaDu cells treated with 5μM menadione or DMSO for 24h and the corresponding control (left panel), percentage of cell cycle phase were calculated (right panel). (C, D, E) METTL3 expression has a positive correlation with MKI67, PCNA and VEGFA in HNSCC (GEPIA website). [file 40164_2022_256_MOESM6_ESM.tif]
